# MALNUTRITION IN COVID-19 SURVIVORS: PREVALENCE AND RISK FACTORS

**DOI:** 10.1093/geroni/igad104.3462

**Published:** 2023-12-21

**Authors:** Matteo Tosato, Riccardo Calvani, Francesco Landi

**Affiliations:** Università Cattolica del Sacro Cuore, Rome, Lazio, Italy; Università Cattolica del Sacro Cuore, Rome, Lazio, Italy; Università Cattolica del Sacro Cuore, Rome, Lazio, Italy

## Abstract

Nutritional status is a critical factor throughout COVID-19 disease course. Malnutrition is associated with poor outcomes in hospitalized COVID-19 patients. The aim of our study is to assess the prevalence of malnutrition and identify its risk factors in COVID-19 survivors. Study cohort included 1230 COVID-19 survivors aged 18-86 attending a post-COVID-19 outpatient service. Data on clinical parameters, anthropometry, acute COVID-19 symptoms, lifestyle habits were collected through a comprehensive medical assessment. Malnutrition was assessed according to Global Leadership Initiative on Malnutrition (GLIM) criteria. Our results showed that the prevalence of malnutrition was 22% at 4-5 months after acute disease. Participants who were not hospitalized during acute COVID-19 showed a higher rate of malnutrition compared to those who needed hospitalization (26% versus 19%, p< 0.01). Malnutrition was diagnosed in 25% COVID-19 survivors over 65 years of age compared to 21% younger participants (p< 0.01). After multivariable adjustment, the likelihood of being malnourished increased progressively and independently with advancing age (Odds ratio [OR] 1.02; 95% CI 1.01–1.03) and in male participants (OR 5.56; 95% CI 3.53–8.74). Malnutrition was associated with loss of appetite (OR 2.50; 95% CI 1.73–3.62), and dysgeusia (OR 4.05; 95% CI 2.30–7.21) during acute COVID-19. In the present investigation we showed that malnutrition was highly prevalent in a large cohort of COVID-19 survivors at 4-5 months from acute illness. Our findings highlight the need to implement comprehensive nutritional assessment and therapy as an integral part of care for COVID-19 patients

